# Comparison of endothelial progenitor cell function in type 2 diabetes with good and poor glycemic control

**DOI:** 10.1186/1472-6823-10-5

**Published:** 2010-04-07

**Authors:** Worachat Churdchomjan, Pakpoom Kheolamai, Sirikul Manochantr, Pirath Tapanadechopone, Chairat Tantrawatpan, Yaowalak U-pratya, Surapol Issaragrisil

**Affiliations:** 1 Division of Cell Biology, Department of Preclinical Sciences, Faculty of Medicine, Thammasat University, Pathumthani 12120, Thailand; 2 Division of Hematology, Department of Medicine, Faculty of Medicine Siriraj Hospital, Mahidol University, Bangkok 10700, Thailand

## Abstract

**Background:**

Endothelial progenitor cells (EPCs) play an important role in vascular repair and a decrease in the number of EPCs is observed in type 2 diabetes. However, there is no report on the change of EPCs after glycemic control. This study therefore aimed to investigate the EPC number and function in patients with good and poor glycemic control.

**Methods:**

The number of EPCs was studied using flow cytometry by co-expression of CD34 and VEGFR2. The EPCs were cultured and characterized by the expression of UEA-I, CD34, VEGFR2, vWF and Dil-Ac-LDL engulfment, as well as the ability to form capillary-like structures. An *in vitro *study on the effect of hyperglycemia on the proliferation and viability of the cultured EPCs was also performed.

**Results:**

The number of EPCs in type 2 diabetes was significantly decreased compared with healthy controls and there was an inverse correlation between the EPC numbers and plasma glucose, as well as HbA1_C_. The number and function of EPCs in patients with good glycemic control were recovered compared with those with poor glycemic control. When glucose was supplemented in the culture *in vitro*, there was a negative effect on the proliferation and viability of EPCs, in a dose-dependent manner, whereas the enhancement of apoptosis was observed.

**Conclusion:**

There was EPC dysfunction in type 2 diabetes which might be improved by strict glycemic control. However, the circulating EPC number and proliferative function in patients with good glycemic control did not reach the level in healthy controls.

## Background

Regeneration and reconstruction of the vascular endothelium is essential in vascular repair processes. This endothelial reconstruction can be accomplished by the proliferation and migration of surrounding mature endothelial cells [1,2]. However, mature endothelial cells are terminally differentiated cells with low proliferative potential, and their capacity to repair damaged vessels is limited [3,4]. Recent studies reveal that endothelial progenitor cells (EPCs) which reside in the bone marrow and to some extent in peripheral blood [5,6], play an important role in angiogenesis through their capacity to proliferate, migrate, differentiate and as a source of paracrine factors for pro-angiogenic cytokines [7,8]. These EPCs co-express surface markers of both hematopoietic stem cells (CD34 and CD133) and endothelial cells (CD146, vWF and VEGFR2, also known as KDR) [9,10].

Previous reports show that the number of circulating EPCs is decreased in both type 1 and type 2 diabetes which is likely to be involved in the pathogenesis of vascular complications [11-13]. These complications can be observed clinically, even in diabetics who achieve good long-term glycemic control [14]. It is therefore possible that there is still EPC dysfunction in diabetics with good glycemic control. We studied the number of the circulating EPCs in patients with type 2 diabetes as divided into good and poor glycemic control groups in order to study the effect of glycemic control. A quantitative assessment of circulating EPCs was made by using flow cytometry. *In vitro *hyperglycemic effect using various glucose concentrations on the viability, proliferation and apoptosis of cultured EPCs from both diabetic patients and healthy controls were also performed.

## Methods

### Subjects

The study was approved by the Ethical Committee and is in accordance with the Helsinki Declaration of 1975. All subjects in the study had given written informed consent prior to participating. The study enrolled 36 patients with type 2 diabetes who attended the diabetic clinic during the period from May to August 2007. Fourteen were male and 22 were female. The ages ranged from 31-86 years (mean 61.5 ± 13.2 years) and the duration of diabetes varied from 1-15 years (mean 6.2 ± 4.3 years). Clinical history and medications were evaluated together with fasting blood sugar (FBS) and glycosylated hemoglobin (HbA1_C_). Patients were divided into two groups according to criteria established by the American Diabetic Association (ADA) [15]; the first group had good glycemic control (FBS ≤ 7.0 mmol/l and HbA1_C _≤ 7.0%) and the second group had poor glycemic control (FBS > 7.0 mmol/l and HbA1_C _> 7.0%). Patients with coronary artery disease, cerebrovascular disease, peripheral vascular disease, chronic inflammation and malignant disease were excluded. There were no significant differences in age, body mass index (BMI), duration of diabetes, blood pressure and lipid profiles, between diabetic patients with good and poor glycemic control (Table 1).

**Table 1:** Baseline characteristics of patients with type 2 diabetes as divided into poor and good glycemic control

	Poor glycemic control	Good glycemic control	*P *value
Number	23	13	-
Age (years)	59.7 ± 13.2 (31-86)	64.7 ± 13.2 (41-86)	0.287
Sex (male:female)	9:14	5:08	-
BMI (kg/m^2^)	23.8 ± 2.8	21.6 ± 4.5	0.516
Duration of diabetes (years)	6.7 ± 4.3 (1-15)	5.2 ± 4.3 (1-13.4)	0.433
Systolic blood pressure (mmHg)	126.2 ± 18 (100-170)	134.4 ± 16 (110-150)	0.251
Diastolic blood pressure (mmHg)	79.1 ± 8.9 (60-90)	76.7 ± 7.1 (70-90)	0.582
HbA1_C _(%)	9.2 ± 1.4 (7-12.2)	6.2 ± 0.5 (5.3-6.6)	< 0.001
FBS (mmol/l)	8.9 ± 1.8 (5.8-11.8)	6.7 ± 0.8 (5.6-8.2)	< 0.001
Total cholesterol (mg/dl)	175.9 ± 31.9 (134-242)	191.8 ± 42.6 (148-264)	0.314
LDL cholesterol (mg/dl)	98.6 ± 25.3 (61-146)	125 ± 41.9 (76-215)	0.936
HDL cholesterol (mg/dl)	51.8 ± 12.8 (35-75)	48 ± 13.6 (32-73)	0.977
Triglyceride (mg/dl)	137.8 ± 70 (54-310)	218.5 ± 121.5 (100-340)	0.124
Therapy			
Statin [n (%)]	15 (65)	7 (53)	-
ACEI/ARB [n (%)]	4 (17)	3 (23)	-
Aspirin [n (%)]	1 (4)	1 (7)	-
OHA [n (%)]	20 (87)	10 (77)	-
Insulin [n (%)]	1 (4)	1 (7)	-
OHD + Insulin [n (%)]	2 (8)	-	-

Data are presented as mean ± SD, percentage or median (interquartile range); ACEI, angiotensin-converting enzyme inhibitor; ARB, angiotensin II receptor blockers; BMI, body-mass index; FBS, fasting blood sugar; HbA1_C_, glycosylated hemoglobin; HDL, high-density lipoprotein; LDL, low-density lipoprotein; OHA, oral hypoglycemic agent.

Twenty healthy medical personnel served as controls. Of these, 5 were male and 15 were female. The ages ranged from 39-83 years (mean 58.1 ± 11.3 years). All controls had no clinical history of diabetes or hyperlipidemia, and had normal FBS of < 7 mmol/l. They had normal physical examination and had not received any medication.

### Isolation and quantification of circulating EPCs

Twenty ml of heparinized blood was used to isolate and quantify the circulating EPCs. Briefly, mononuclear cells (MNCs) were isolated using Ficoll-Paque (Amersham Bioscience, USA) density gradient centrifugation (400 *g*, 20°C, 20 minutes), then washed twice with phosphate buffered saline (PBS), counted, and resuspended in PBS. Quantification of circulating EPCs was performed by flow cytometry as previously described [16]. Briefly, 1 × 10^6 ^MNCs were incubated with a combination of a FITC-conjugated anti-human CD34 antibody (BD Biosciences, USA) and a PE-conjugated anti-human VEGFR2 antibody (R&D Systems, USA) for 15 minutes at 4°C in the dark. Cell pellets were washed twice with PBS and fixed with 2% (v/v) paraformaldehyde in PBS. The preparations were kept at 4°C in the dark. Flow cytometry analysis was performed using a FACScalibur™ flow cytometer (Becton Dickinson, USA) and the CellQuest^® ^FACs software.

### Culture and characterization of circulating EPCs

One million MNCs were resuspended in endothelial cell growth medium [endothelial basal medium-2 (EBM-2) (Clonetics; Walkersville, USA), supplemented with EGM-2 single aliquots containing 2% (v/v) fetal bovine serum, 5 μg/ml epidermal growth factor, 200 μg/ml hydrocortisone, 0.5 μg/ml vascular endothelial growth factor, 10 μg/ml basic fibroblast growth factor, 20 μg/ml long R3 insulin-like growth factor 1, and 1 mg/ml ascorbic acid]. They were then plated in a T-25 culture flask coated with 10 μg/ml human fibronectin (Amersham Biosciences, USA) and after culture for 3 days, non-adherent cells were removed and fresh medium was added. Thereafter, the medium was replaced every 3 days for the entire culture period. Identification of EPCs was performed by staining with 1,1-dioctadecyl-3,3,3,3-tetramethylindocarbocyanine-labeled Ac-LDL (Dil-Ac-LDL) and FITC-conjugated *Ulex europaeus *agglutinin-I (UEA-I) as described previously [7,16]. Briefly, cells were cultured on coverslips coated with 10 μg/ml human fibronectin (Amersham Biosciences, USA). At the required time points, medium was removed from plates and the adherent cells on coverslips were incubated with 10 μg/ml Dil-Ac-LDL (Bio Technologies, USA) at 37°C for 4 hours. The cells were then washed three times with PBS, fixed with 2% (v/v) paraformaldehyde in PBS, incubated with 200 μL mouse anti-human UEA-I antibody-conjugated with FITC (Sigma, USA) at 4°C for 1 hours, and then washed three times with PBS. All slides were kept in a light-tight box and examined by fluorescence microscopy.

The cultured EPCs were also incubated with a FITC-conjugated anti-human CD34 antibody (BD Biosciences, USA), a PE-conjugated anti-human VEGFR2 antibody (R&D Systems, USA), a FITC-conjugated anti-human CD146 antibody (BD Biosciences, USA), and a FITC-conjugated anti-human vWF antibody (R&D Systems, USA) for 15 minutes at 4°C in the dark. Cell pellets were washed twice with PBS and fixed with 2% (v/v) paraformaldehyde in PBS. The preparations were kept at 4°C in the dark. Flow cytometry analysis was performed using a FACScalibur™ flow cytometer (Becton Dickinson, USA) and the CellQuest^® ^FACs software.

### Formation of capillary-like structures on Matrigel

Twenty-four well plates were coated with 500 μL of Matrigel (BD Biosciences, USA) and 4 × 10^4 ^EPCs were added to each well. The plates were incubated for 24 hours and examined by light microscopy for the ability to form capillary-like structures.

### In vitro studies on the effects of hyperglycemia on cultured EPCs

The effects of various glucose concentrations on cultured EPCs from both diabetic patients and healthy controls were studied for viability, proliferation, and apoptosis. Passage four EPCs were used as target cells. Cells samples were cultured in endothelial cell growth medium supplemented with various concentrations of D-glucose at final concentrations of 7.8, 10.5, 13.5, 16.5 and 19.5 mmol/l.

#### (a) Effect on viability

Viability of cells was studied using trypan blue staining after 21 days of culture. Cultured cells from each treatment group were trypsinized and the number of viable cells was counted using a hemacytometer. The results were compared with controls of EPCs cultured in 5.5 mmol/l of D-glucose.

#### (b) Effect on proliferative function

The influence of increasing concentrations of glucose on the proliferative capability of cultured EPCs was assessed using the colorimetric MTT [3-(4,5-dimethylthiazol-2-yl) K2,5-diphenyl tetrazolium bromide] proliferation assay (Sigma, USA) [17]. Briefly, 1 × 10^4 ^cultured EPCs were seeded into each well of a 96-well plate containing 100 μl endothelial cell growth medium. Cell samples were then exposed to various concentrations of D-glucose as described previously. After 7, 14 and 21 days of continuous exposure, 1 mg/ml of MTT reagent was added to each well and the cells were incubated for another 4 hours at 37°C. The absorbance was measured at 595 nm using a spectrophotometer. The data were presented as a percent of proliferative inhibition, which was calculated by the following formula:

#### (c) Effect on apoptosis

The percentage of apoptotic cells was studied by flow cytometry using a 7-AAD/annexin V apoptosis detection kit (BD Biosciences, USA) [18]. Briefly, 2 × 10^5 ^cultured EPCs were seeded into each well of a 24-well plate containing 1 ml endothelial cell growth medium. Cell samples were then exposed to various concentrations of D-glucose as previously described. After culture for 7 and 14 days, the cells were harvested and washed twice with PBS. They were then resuspended in binding buffer, supplemented with 5 μl annexin V and 5 μL 7-AAD, at 2 × 10^5 ^cells/100 μl binding buffer and incubated at room temperature for 15 minutes in the dark. Flow cytometry analysis was performed using a FACScalibur™ (Becton Dickinson, USA) and CellQuest^® ^FACs software.

### Statistical analysis

Data are presented as mean ± SD or mean ± SEM as indicated. For comparison, unpaired Student t-tests or ANOVA tests were used. The relationship was calculated using Spearman's correlation coefficient. A *P *value of < 0.05 was considered to be statistically significant.

## Results

### Number of circulating EPCs

**Figure 1:**
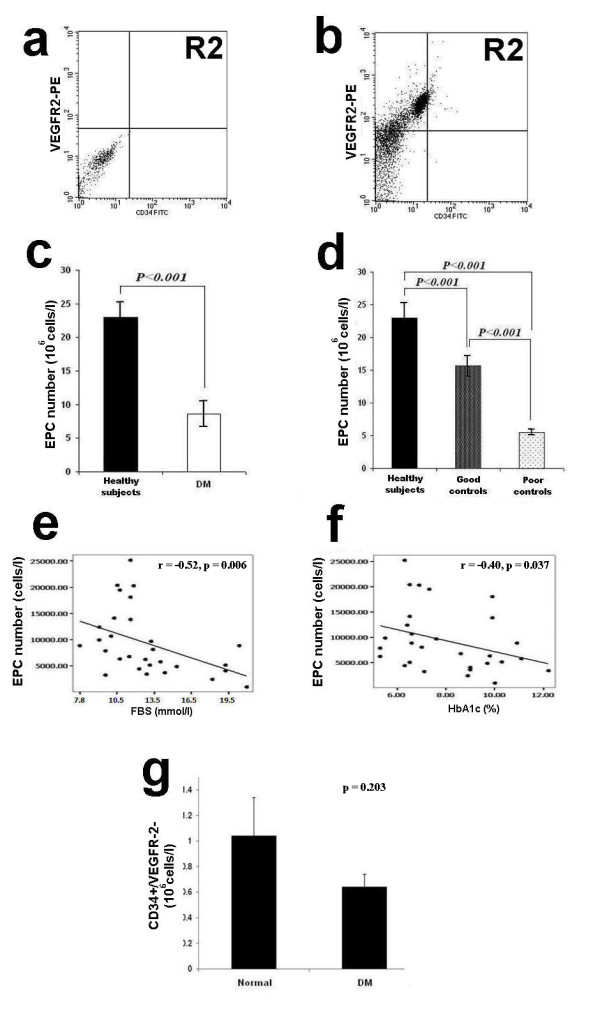
**Circulating EPC number in healthy controls and diabetes**. **a **and **b**: Circulating EPC numbers were determined by flow cytometry for the co-expression of CD34 and VEGFR2 (**b**). Peripheral blood MNCs incubated with IgG isotype control (**a**) serve as a negative control to determine the intrinsic fluorescent intensity of the peripheral blood MNCs and to define positive area R2. **c**: Absolute number of circulating EPCs in healthy controls and diabetes as determined by the co-expression of CD34 and VEGFR2. **d**: Absolute number of circulating EPCs in healthy controls, diabetes with good and poor glycemic control as determined by the co-expression of CD34 and VEGFR2. **e**: Correlation between the circulating EPC numbers and FBS, **f**: Correlation between the circulating EPC numbers and HbA1_C_, **g: **Absolute number of circulating CD34+/VEGFR2- cells in healthy controls and diabetes. Data are presented as means ± SEM.

The numbers of circulating EPCs, as determined by co-expression of the two EPC markers, CD34 and VEGFR2, and quantified using flow cytometry, are shown in Fig. 1a-b. Fig. 1c illustrates absolute number of circulating EPCs in diabetic patients in comparison with those in healthy controls. The absolute number of circulating EPCs was significantly lower in diabetic patients compared with healthy controls (8.6 × 10^6 ^± 5.9 × 10^6 ^vs. 23 × 10^6 ^± 2.3 × 10^6^, *P *< 0.001). Notably, the absolute number of circulating EPCs in diabetic patients with good glycemic control was significantly higher than that of poor glycemic control (15.6 × 10^6 ^± 1.6 × 10^6 ^vs. 5.5 × 10^6 ^± 0.5 × 10^6^, *P *< 0.001), but still significantly lower than healthy controls (15.6 × 10^6 ^± 1.6 × 10^6 ^vs. 23 × 10^6 ^± 2.3 × 10^6^, *P *< 0.001) (Fig. 1d). In diabetic patients, Spearman's correlation analysis revealed that the numbers of circulating EPCs were inversely correlated with the concentrations of both fasting blood sugar (*r *= -0.52, *P *< 0.05) and HbA1_C _(*r *= -0.40, *P *< 0.05) (Fig. 1e, f).

### Characterization of cultured EPCs

**Figure 2:**
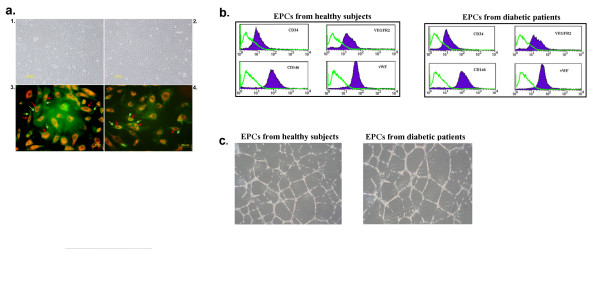
**Characteristics of isolated EPCs from healthy controls and diabetes**. **a1 **and **a2**: Phase contrast microscopic photos of representative cultured EPCs (passage 2) isolated from MNCs of normal healthy controls (**a1**) and MNCs of diabetic patients (**a2**). **a3 **and **a4: **Fluorescent microscopic photos of representative cultured EPCs isolated from MNCs of normal healthy controls (**a3**) and MNCs of diabetic patients (**a4**). Red color (indicated by red arrows) represents fluorescent staining of DiI-Ac-LDL and green color (indicated by green arrows) represents fluorescent staining of FITC-conjugated UEA-I. [Fig. a1 and a2, Magnification × 200; Fig. a3 and a4, Magnification × 400], **b**: Representative histogram based on flow cytometric analysis immunolabelling with a control antibody (green line) and specific antibodies (blue line) to EPC-related markers (CD146, CD34, VEGFR2 and CD146), **c**: EPCs from both healthy subjects and diabetic patients formed capillary-like structure on Matrigel [Magnification × 40].

Following the culture of MNCs in endothelial cell growth medium for at least 3 weeks, the EPC colonies were observed. Those isolated from both diabetic patients and healthy controls displayed a similar morphology, being spindle-shaped cells with a low nuclear/cytoplasmic ratio (Fig. 2a). EPCs isolated from both sources also displayed characteristic EPC phenotypes by uptaking Dil-Ac-LDL (Fig. 2a), expressing UEA-I (Fig. 2a), CD34, vWF, CD146 and VEGFR2 on the plasma membrane (Fig. 2b), as well as the ability to form capillary-like structures on Matrigel (Fig 2c). However, culture of MNCs from diabetic patients took a significantly longer period of time to form colonies with cobblestone appearance (EPC-like colonies) than healthy controls (30.8 ± 3.9 days vs. 22.4 ± 2.7 days, *P *< 0.05).

### In vitro study on the effect of hyperglycemia on cultured EPCs

#### (a) Effect on viability

**Figure 3:**
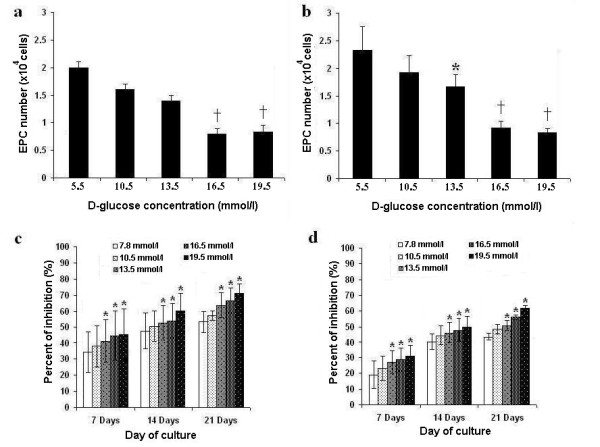
**Number and proliferative capacity of EPCs cultured in hyperglycemic conditions**. **a **and **b**: Effect of various glucose concentrations on the number of cultured EPCs isolated from healthy controls (**a**) and diabetes (**b**) after 21 days of culture. **c **and **d**: Effect of various glucose concentrations on the proliferative capacity of cultured EPCs isolated from diabetes (**c**) and healthy controls (**d**) as determined by MTT assay. * *p *< 0.05, † *p *< 0.01 versus control.

The effects of hyperglycemia on EPC viability were studied by exposing cultured EPCs to various concentrations of D-glucose for 21 days as shown in Fig. 3a,b. The number of cultured EPCs in high glucose concentrations (13.5, 16.5 and 19.5 mmol/l) was significantly reduced compared with controls (EPCs cultured in 5.5 mmol/l of D-glucose) (*P *< 0.05), and in a dose-dependent manner.

#### (b) Effect on proliferative function

The effects of hyperglycemia on proliferative function of EPCs determined by a MTT assay are shown in Fig. 3c,d. The proliferation of cultured EPCs from both diabetic patients and healthy controls decreased steadily in a dose- and time-dependent manner in response to increasing concentrations of glucose. The proliferation of cultured EPCs was significantly inhibited even in the glucose concentration corresponding to the diabetes with good glycemic control (7.8 mmol/l), compared with controls (EPCs cultured in 5.5 mmol/l of D-glucose) (Fig. 3c,d) (*P *< 0.05). These findings indicate that hyperglycemia had a negative effect on the proliferative capacity of EPCs in a dose-dependent and time-dependent manner.

#### (c) Effect on apoptosis

**Figure 4:**
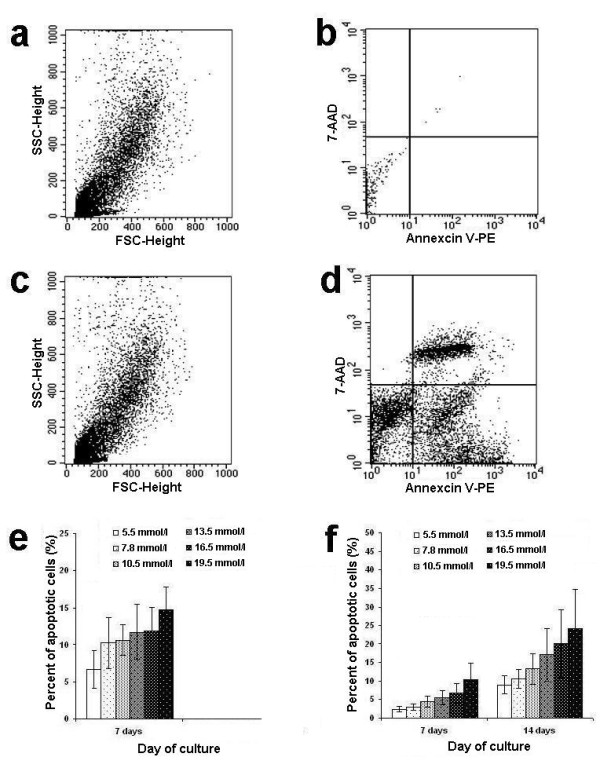
**Apoptotic rate of EPCs cultured in hyperglycemic conditions**. **a-d**: Apoptotic EPCs as identified by flow cytometry with the expression of annexin V and 7-AAD. **e **and **f**: Effect of various glucose concentrations on the apoptotic rate of cultured EPCs from diabetes (**e**) and healthy controls (**f**) after 14 days of culture.

The effects of hyperglycemia on apoptosis of EPCs were determined using flow cytometry, as shown in Fig. 4a-d. The apoptotic rates of cultured EPCs were significantly increased with high glucose concentrations in a dose- and time-dependent manner compared with controls (EPCs cultured in 5.5 mmol/l of D-glucose) (Fig. 4e,f). At a glucose concentration of 7.8 mmol/l, the EPC apoptotic rate was still increased compared with controls (Fig. 4e,f). There were no viable cultured EPCs from diabetic patients after 14 days of culture, so the apoptotic rate could not be studied (Fig. 4f).

## Discussion

Previous attempts to culture EPCs gave rise to highly variable results due, primarily to divergent culture conditions [11,19-21], and in addition, the absolute number of EPCs could not be quantified due to the lack of EPC-related cell markers. Recently, the identification and quantification of circulating EPCs using antibodies against EPC-related cell surface antigens and flow cytometric analysis have been developed [22]. This methodology is not only more sensitive and specific but also makes possible the quantification of circulating EPCs. However, even with the advent of this new technology, there have been only a few reports using this methodology to identify and quantify the number of circulating EPCs in diabetic patients [22-24].

A reduction in EPC number is likely to influence vascular integrity as it was reported that the EPC number is a surrogate marker for vascular function and cumulative cardiovascular risk in healthy persons [6,19,25-28]. In diabetic patients with vascular complications, there was a marked reduction of circulating EPCs compared with those without vasculopathy and the EPC numbers correlated with the degree of severity of the vascular complications [22]. Our observations that the EPC numbers were significantly higher in diabetic patients with good glycemic control compared with those with poor glycemic control, supports our hypothesis that the EPC function can be improved by strict control of blood glucose to a normal level, and thereby preventing or ameliorating the development of severe vascular complications in diabetic patients.

A reduction of circulating EPCs in diabetic patients may reflect a shortened peripheral survival of EPCs, or a poor mobilization of EPCs from the bone marrow. Our results show that the absolute number of circulating CD34+ and EPC (CD34+/VEGFR2+) cells in diabetes were significantly lower than healthy controls, whereas the absolute number of CD34+/VEGFR2- in diabetic patients was not different from healthy controls (0.767 ± 0.18 vs. 1.033 ± 0.27, *p *= 0.203) (Fig. 1g). Although this finding suggests that there is a decreased survival of circulating EPCs in diabetic patients, the possibility of poor mobilization of EPCs from the bone marrow cannot be excluded. Previous studies in a rat model of hindlimb ischemia/reperfusion injury, showed that there was a defective EPC mobilization from bone marrow in diabetic rats compared with controls [7]. Furthermore, there was additional evidence in mice that endothelial nitric oxide synthase (eNOS) activation is impaired in diabetes resulting in poor mobilization of EPCs [29].

*In vitro *analysis of the effect of increased glucose concentration on cultured EPCs revealed that hyperglycemia significantly decreased EPC viability and proliferation, and increased apoptosis, in dose-dependent manners. We used various concentrations of glucose ranging from 7.8 mmol/l (equivalent to FBS of 140 mg/dl) to 19.5 mmol/l (equivalent to FBS of 345 mg/dl) to simulate hyperglycemia because these are more or less similar to the conditions in diabetic patients, whereas previous studies had used a higher glucose concentration of 33 mmol/l [30-33]. Although D-glucose can increase osmotic stress which may directly affect the EPCs, the previous studies showed that hyperosmolarity using both L-glucose and mannitol had no effect on EPCs [34] indicating that hyperosmolarity has no effect on EPCs.

The mechanism contributing to the reduction of circulating EPCs in diabetes is still unknown, however, it may be due to decreased proliferation and/or accelerated cell death. Previous studies showed that exposure to hyperglycemic conditions resulted in an accumulation of EPCs in resting stage and decreased number of EPCs in proliferative stage [34]. This finding indicated an up-regulation of p16 and p21 involved in the regulation of G1-S phase transition and a decreased number of proliferating EPCs in hyperglycemia. Further studies have shown that an altered expression level of p16 and p21 is associated with apoptosis in several cell types [34-36]. In addition, hyperglycemia can up-regulate the expression of ETS transcription factor which plays an important role in proliferation, survival and differentiation, resulting in the blockade of the functional activity of EPCs [37]. Moreover, increased oxidative stress, as shown by increased production of free radicals and/or impaired anti-oxidant defense capabilities, were reported in patients with diabetes [38,39]. Also, there was a report indicating a relationship between various disturbances in mitochondrial function and diabetes [40]. However, the EPCs are known to be relatively resistant to oxidative stress than the mature endothelial cells [41]. It is still uncertain that the presence of oxidative stress may contribute to the impaired EPC function/proliferation in high glucose status [42].

The reduced viability and proliferation of EPCs in the hyperglycemic condition might also involve the phosphatidylinositol 3'-kinase (PI3k)/serine-threonine kinase (Akt)/endothelial nitric oxide synthase (eNOS) pathway [PI3k/Akt/eNOS]. Previous reports indicate that activating the PI3k/Akt in endothelial cells can prevent cell death [43], resulting in increasing cell survival and activating eNOS which leads to NO production [44]. Recent studies show that the PI3k/Akt/eNOS pathway plays an important role in preventing high glucose-induced cell injury [45]. It is likely that hyperglycemia inhibits the PI3k/Akt/eNOS pathway in EPCs, thus resulting in our observed reduced viability and proliferation, with increased EPC apoptosis.

Our findings that the EPC number in patients with good glycemic control was significantly higher than those with poor glycemic control, and when more glucose was added to EPC cultures *in vitro*, viability and proliferation decreased whereas apoptosis increased, indirectly supports the importance of strict control of blood glucose in preventing vascular complications.

However, the observed circulating EPC number in patients with good glycemic control did not reach the level found in healthy controls. As reported previously [46], there appears to be some degree of vascular damage which is irreversible, even after strict glycemic control. Additional strategies to further improve the EPC number and functions in diabetic patients are required. However, our observations were obtained from a cross-sectional study, so it should be confirmed in a cohort study with longitudinal follow-up, to determine whether strict glycemic control can improve EPC functions and thereby prevent vascular complications.

## Conclusion

There was EPC dysfunction in type 2 diabetes which might be improved by strict glycemic control. The circulating EPC number and proliferative function in patients with good glycemic control did not reach the level in healthy controls. However, these observations obtained from a cross-sectional study should be confirmed in a cohort study.

## Abbreviations

**Akt**: serine/threonine kinase; **BMI**: body mass index; **DiI-Ac-LDL**: Dil-labeled acetylated low density lipoprotein; **DM**: diabetes mellitus; **EBM-2**: endothelial basal medium-2; **eNOS**: endothelial nitric oxide synthase; **EPC**: endothelial progenitor cell; **FBS**: fasting blood sugar; **FITC**: flourescein isothiocyanate; **HbA1c**: glycosylated hemoglobin; **HDL**: high density lipoprotein; **HUVEC**: human umbilical vein endothelial cells; **MNCs**: mononuclear cells; **MTT**: 3-(4,5-dimethylthiazol-2-yl) K2,5-diphenyl tetrazolium bromide; **NO**: nitric oxide; **PBS**: phosphate buffered saline; **PE**: phycoerythrin; **PI3k**: phosphatidylinositol 3'-kinase; **UEA-I**: Ulex europaeus agglutinin-I; **VEGF**: vascular endothelial growth factor; **VEGFR2**: vascular endothelial growth factor receptor-2; **vWF**: von Willebrand factor.

## Competing interests

The authors declare that they have no competing interests.

## Authors' contributions

WC carried out the experiments, performed data analysis and drafted the manuscript. PK performed data analysis, supervised the study and drafted the manuscript. SM supervised the study and drafted the manuscript. CT drafted the manuscript. YU carried out the experiments and performed data analysis. SI designed, supervised the study and finalised the manuscript. All authors read and approved the final manuscript.

## Pre-publication history

The pre-publication history for this paper can be accessed here:

http://www.biomedcentral.com/1472-6823/10/5/prepub
